# The success of the tumor immunotherapy: neutrophils from bench to beside

**DOI:** 10.3389/fimmu.2025.1524038

**Published:** 2025-01-24

**Authors:** Meng Zhu, Ru Jia, Xiaojie Zhang, Pingwei Xu

**Affiliations:** ^1^ The First Clinical Medical College, Wenzhou Medical University, Wenzhou, Zhejiang, China; ^2^ Department of Obstetrics, The First Affiliated Hospital of Wenzhou Medical University, Wenzhou, Zhejiang, China; ^3^ Translational Medicine Laboratory, The First Affiliated Hospital of Wenzhou Medical University, Wenzhou, Zhejiang, China

**Keywords:** neutrophils, tumor immunotherapy, tumor cell-derived microparticles, immune checkpoint, innate immunity

## Abstract

The present immune therapy was focused on the immune checkpoint blockade or Chimeric Antigen Receptor T-Cell Immunotherapy (CART) transfer, but how to activate the innate immune system to antitumor still lags out. Neutrophils are the most abundant circulating leukocytes in human, and heterogeneous neutrophils have been increasingly recognized as important players in tumor progression. They play double “edge-sward” by either supporting or suppressing the tumor growth, including driving angiogenesis, extracellular matrix remodeling to promote tumor growth, participating in antitumor adaptive immunity, or killing tumor cells directly to inhibit the tumor growth. The complex role of neutrophils in various tumors depends on the tumor microenvironment (TME) they are located, and emerging evidence has suggested that neutrophils may determine the success of tumor immunotherapy in the context of the immune checkpoint blockade, innate immune training, or drug-loaded extracellular microvesicles therapy, which makes them become an exciting target for tumor immunotherapy, but still with challenges. Here, we summarize the latest insights on how to activate neutrophils in antitumor immunity and discuss the advances of neutrophil-targeted immunotherapy strategies.

## Introduction

Neutrophils, the most abundant leukocytes in human peripheral blood circulation, have traditionally been recognized as the frontline defenders against pathogenic microorganisms. Our body produces 10^11^ neutrophils every day, most of which die without activation (1, 2). The circulation neutrophils with a short lifespan about 6-8 hours (3). It has been reported that neutrophils have an increased life span when activated or entering tissues, which can survive 5.4 days in humans and 6.5 days in mice (4). In a mouse model of head and neck cancer, intravital multiphoton imaging showed that neutrophils could survive for 3 days in tumor microenvironment (TME) (5). Meanwhile, *in vitro* investigations have revealed that tumor cells secrete cytokines, including granulocyte colony-stimulating factor (G-CSF), GLUT1, or TNF, which can prolong the lifespan of neutrophils and bolster their viability (6–9).

Although their role in host defense has been confirmed, their role in tumor biology remains to be elucidated. The neutrophils influence the immune status in the TME, they can be retrained to help the tumor growth directly, or indirectly by suppressing the antitumor responses of T-cells and macrophages. They generate cytotoxic substances such as proteases, defensins, and reactive oxygen species (ROS) or reactive nitrogen species (RNS) to directly promote tumor cell apoptosis (10, 11). Emerging evidence suggests that activating neutrophils has therapeutic effects in tumor immunotherapy, including the Bacillus Calmette-Guérin (BCG), β-glucan, or a combination therapy involving anti-CD40 antibodies, TNF-α, and tumor-binding antibodies (12–14). Moreover, the activated neutrophils can eradicate the tumor antigen-lost tumor cells in T cell-based immunotherapies. Extracellular vesicles, including exosomes (30-50 nm in diameter) and microvesicles (shedding from the membrane, 100-1000 nm in diameter), were the ideal carriers for chemotherapeutic drugs or mRNA (15–17). Tumor cell-derived microvesicles that packaged chemotherapeutic drugs are a novelty strategy in clinical use in the malignant pleural effusion (MPE) and cholangiocarcinoma (18–20). In this paper, we summarize the mechanism by which neutrophils inhibit tumor progression and the latest advances in targeting neutrophils in tumor immunotherapy.

## Characteristics of tumor-associated neutrophils

Neutrophils undergo differentiation and maturation under the influence of G-CSF before entering the circulation (2, 9, 21). They originate from common myeloid progenitors (CMPs), starting from granulocyte monocyte progenitors (GMPs), and go through a series of maturation stages, from the mitotic neutrophil pool (including myeloblasts, promyelocytes, and myelocytes) to metamyelocyte, and finally differentiate into polymorphonuclear neutrophils (PMNs) (22, 23). During this differentiation process, the promyelocyte precursor’s circular nucleus undergoes significant morphological transformations, including nuclear segmentation and the accretion of peripheral heterochromatin (21, 24), which are different in humans and mice (25). The recently discovered human CD66b^-^CD64^dim^CD115^-^ neutrophil-committed progenitor cells (NCPs) in the bone marrow are more immature in transcriptome and phenotype compared to GMPs (26). A systematic study was conducted on NCPs using a combination of flow cytometry and single-cell multi-omics techniques, and it was found that there are four subpopulations of cells at different stages of maturation in NCPs, which can be selectively differentiated into CD66b^+^ neutrophils through two different differentiation pathways (26). The lifespan of neutrophils is very short, about 6-8 hours (3, 27), during which the total number of cells oscillates in a circadian rhythm. In mice, neutrophils are released from the bone marrow into the bloodstream at night. At the end of the night, aging neutrophils return to the bone marrow and are cleared by macrophages (28). At this time, they occupy the ecological niche of other cells such as hematopoietic stem cells, and promote the release of hematopoietic stem cells by regulating adhesion molecules such as CXCL12 (29). The diurnal migration of neutrophils may be a feedback mechanism by which bone marrow stromal cells perceive the concentration of white blood cells in the blood and trigger the next cycle of hematopoietic cell release.

It is known that in early life, the gut microbiota interacts with the developing immune system and undergoes immune training (30). The microbiota actively regulates the production of neutrophils by releasing various bacterial cell components, secreting factors, and metabolites. The strongest evidence is that the use of antibiotics or in a sterile state reduces the number of neutrophils and their precursors in the bone marrow, spleen, and circulation of mice (31), while E. coli strains and cecal contents of mice can restore the number of neutrophils (32). Neutrophils are attracted to the intestinal lumen through the high-affinity formyl peptide receptor 1 (FPR1), indicating an important interaction between the microbiota and neutrophil migration (33). The gut microbiota can also enhance neutrophil recruitment in a TLR4-dependent manner (34). Metabolites produced by microbial communities, such as short-chain fatty acids, indole derivatives, secondary bile acids, etc., have been shown to directly mature neutrophils and regulate their function (35). For example, butyrate can affect immune responses through G protein-coupled receptors (GPCRs) and histone deacetylases (36). The gut microbiota can also control the maturation and aging of neutrophils through the TLR and MYD88 pathways (37).

Similar to macrophages, neutrophils have been artificially classified into N1 antitumor and N2 pro-tumor subsets (38). In humans, neutrophils were marked by CD11b^+^CD15^+^CD49d^-^, while in mice, the neutrophils were marked by CD11b^+^Ly-6G^+^. N1 neutrophils exhibit a pro-inflammatory phenotype, while N2 neutrophils, marked by immature band or ring nucleus, display a pro-tumorigenic profile (6, 22, 39). In addition, N1 and N2 neutrophils can be divided by density, the low-density neutrophil (LDN), the normal-density neutrophil (NDN), and the high-density neutrophil (HDN). The LDNs are immature neutrophils with a band of ring nuclei, the HDNs are the mature neutrophils with the hyper-segment nucleus (40). LDN may have higher proliferation potential and lower maturation status. Recent studies have revealed that LDNs isolated from the peripheral blood of cancer patients, pregnant women, or patients with infectious diseases have immunosuppressive functions (41). In stress response, the proportion of LDN increases, and these cells exhibit lower expression of mature markers such as CD11b and CD16. LDNs may play an important role in inflammatory responses due to their high ability to release inflammatory mediators. NDN mainly includes mature neutrophils, which exhibit high maturity and functional activity in stress response, and are more focused on immediate immune defense (9).

Due to the influence of the surrounding environment, the function of neutrophils has plasticity (42). The N1 neutrophils can be polarized to N2 neutrophils in the presence of TGF-β, while the N2 neutrophils can also be polarized to N1 neutrophils in the presence of interferon-β or a combination of interferon-γ and GM-CSF (38, 43). Smad3 is a regulatory factor for neutrophil polarization within TME. The enhanced Smad3 signaling promotes neutrophil polarization towards N2 phenotype, while the lack of Smad3 in neutrophils leads to their polarization towards antitumor phenotype (44).

## How neutrophils recruitment to TME

The recruitment of neutrophils to the TME is a multifaceted process that involves the interplay of various signaling molecules and cellular interactions. CXCL1, CXCL2, GM-CSF, and IL-1β were well known for neutrophil chemotaxis (45–47). In humans, CXCL8, also known as IL-8, has the strongest chemotactic ability (48–50). Leukotrienes B4, C5a, and PGE2 also can recruit neutrophils to the TME (22, 51, 52). Furthermore, lactic acid, the terminal product of glycolysis, was reported to recruit the neutrophils and induce the neutrophils to increase the PD-1 expression (16), leading to selective attraction of the N1 phenotype to TME. The neutrophils from the bone marrow into the peripheral circulation are governed by the CXC-chemokine receptor 2 (CXCR2) and CXCR4 (2, 53). The reduction in CXCR4 signaling combined with the activation of CXCR2 signaling elicits the neutrophils’ entry into the peripheral circulation (54). The process of neutrophil migration from blood vessels to specific sites is artificially divided into four stages: rolling, adhesion, crawling, and transmigration (53).

## The means by which neutrophil fulfill their function

### Phagocytosis

**Figure 1:**
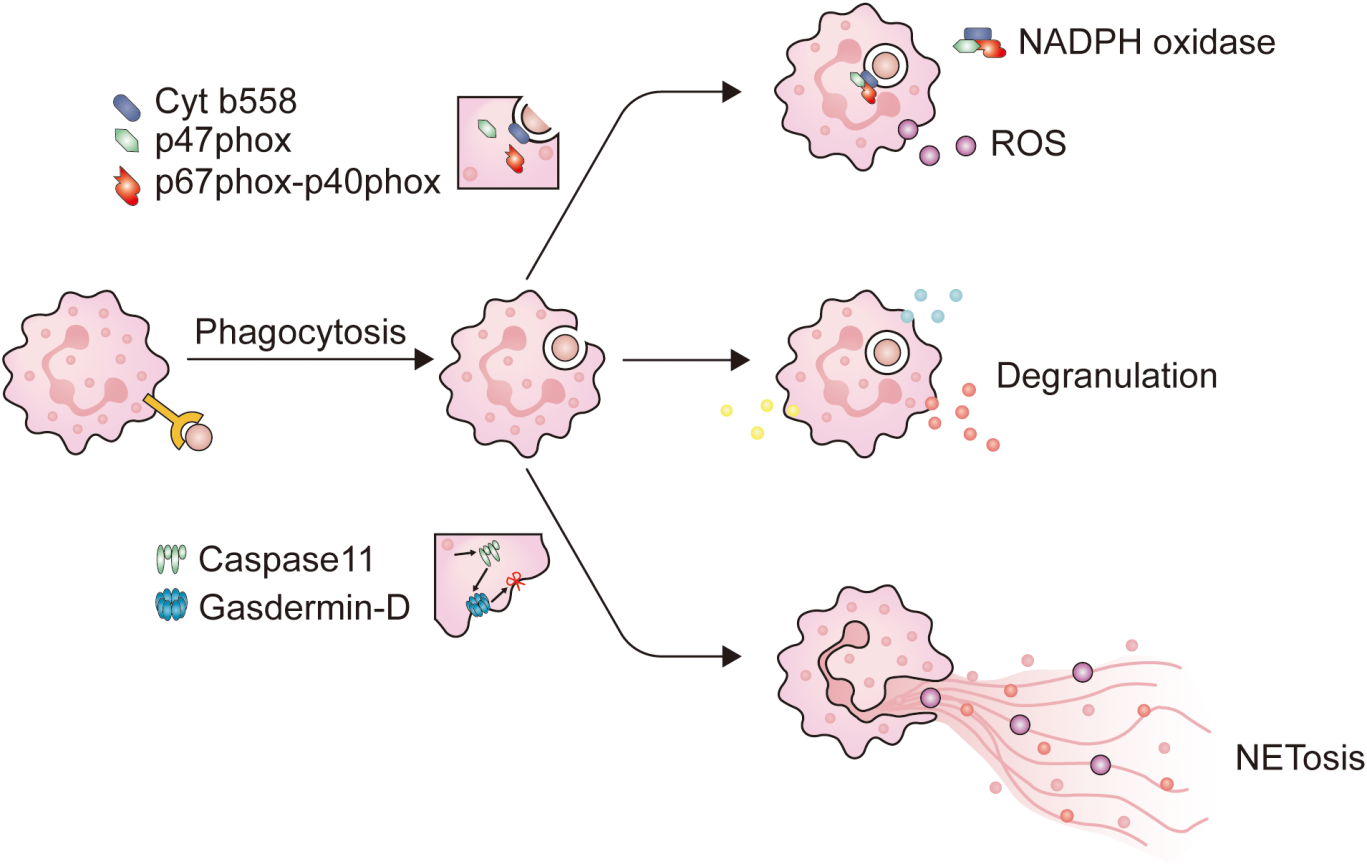
The main mechanism of neutrophils in combating pathogens. When neutrophils come into contact with opsonized pathogens, phagocytosis is initiated. Firstly, neutrophils extend pseudopodia and encapsule the immunological synapse from all directions, until the immunological synapse detaches from the plasma membrane to form phagosomes. When neutrophils form phagosomes, a portion of the plasma membrane invaginates, causing cytochrome b558 on the plasma membrane to bind with intracellular p47phox and p67phox-p40phox, and assemble into NADPH oxidase. NADPH oxidase is a key enzyme for intracellular ROS production. At the same time, after entering the cytoplasm, the phagosomes come into contact and fuse with neutrophil granules, triggering neutrophil degranulation. NET is a filamentous extracellular structure formed by activated neutrophils. NET formation relies on ROS, MPO, and NE to degrade histones, causing chromosome depolymerization. NE will also activate Caspase11, which binds to Gasdermin-D on the plasma membrane, causing the plasma membrane to be incomplete, and NETs are released into the extracellular space to exert their effects.

After reaching the tumor site, neutrophils deploy a series of immune defense strategies. Initially, they initiate receptor-mediated endocytosis, known as phagocytosis. Studies have shown that when target cells are identified as abnormal through antibodies or complement systems, phagocytic cells are primed for optimal function (46, 55). The CR3 and Fc receptors carried by neutrophils mediate complement-dependent cytotoxicity (CDC) and antibody-dependent cytotoxicity (ADCC), respectively (56). The onset of phagocytosis depends on proximity between tumor cells and CD11b/CD18 expressed on neutrophils (57). Opsonized tumor cells contact with CR3 or FcRs and adhere to neutrophils through receptors on the surface of neutrophils (58), ultimately initiating phagocytosis after the formation of immune synapses. The engulfment of tumor cells by neutrophils to form phagosomes requires the involvement of calcium ions (Ca^2+^) and a series of functional proteins (58). After this, neutrophils use various methods to eliminate tumor cells, including degranulation, NETosis, and the release of ROS (**Figure 1**).

Neutrophils internalize fragmented cancer cell plasma membranes and cytoplasmic components through successive bites, eventually precipitating cell necrosis (59, 60). Moreover, it has been observed that C-type lectin receptors (CLRs) on neutrophils can recognize Nidogen-1 on the tumor cell surface, thereby enhancing neutrophil-mediated destruction of these cells (61).

**Figure 2:**
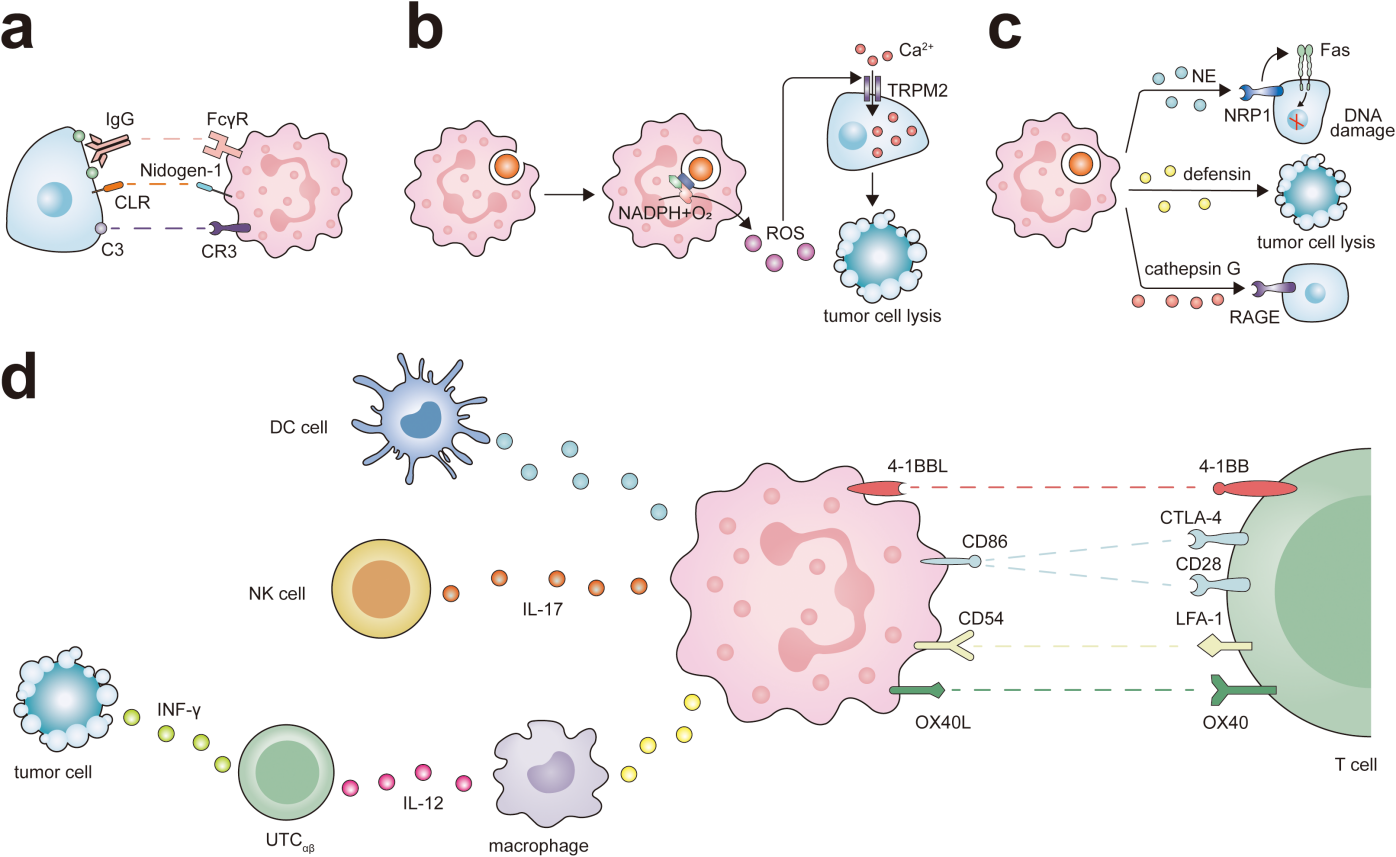
Antitumor potential of neutrophils. Neutrophils exert their different antitumor functions, including direct cytotoxicity against tumor cells and activation of T cell-dependent antitumor immunity. **(A)** Neutrophils express IgG Fc receptors (FcγR) and complement C3 receptor (CR3), which participates in eliminating cancer cells through ADCC or CDC. At the same time, the interaction between CLR and Nidogen-1 plays a role in neutrophil recognition of tumor cells, and this interaction promotes neutrophil-mediated tumor cell killing. **(B)** The ROS generated by neutrophils triggers intracellular signaling pathways in tumor cells, leading to the activation and opening of non-selective transient receptor potential of melistatin 2 (TRPM2), thereby inducing lethal Ca^2+^ influx into cancer cells. **(C)** Neutrophils release various antitumor factors through degranulation. NE via Fas and histone H1 isoforms that are expressed in numerous tumors types, induces apoptosis in cancer cells. Defensins are the most abundant component of the azurophil granules of neutrophils and are highly toxic against several types of tumor cells. the receptor for advanced glycation end products (RAGE) facilitates neutrophil recognition of tumor cells and the RAGE-Cathepsin G interaction is required for neutrophil cytotoxicity. **(D)** Neutrophils can promote the antigen presentation function of DC cells and ADCC of NK cells via IL-17. Neutrophils interact with macrophages and UTC_αβ_ in a tripartite manner. Neutrophils promote macrophage production of IL-12, which in turn promotes the polarization of UTC_αβ_ and the production of IFN-γ. Neutrophils express a set of ligands for lymphocyte checkpoints, including 4-1BBL, CD86, CD54, and OX40L representing potential targets to activate the process of neutrophil-mediated T cell antitumor responses.

The activation of neutrophils is caused by signals emitted by inflammation or infectious lesions, leading to the release of cytotoxins and activation of phagocytosis. Throughout this cascade reaction, neutrophils increased the expression of CD11b, CD18, and CD66b, which are stored within granules (40, 62). In certain circumstances, CD177 and proteinase 3 (PRTN3) are also appreciably upregulated (63). Unlike the conventionally activated neutrophils found in the peripheral blood, those neutrophils that infiltrate into tumors exhibit a distinct activated phenotype, characterized by the increased expression of CD54 (40). After activation, neutrophils predominantly fulfill their function through the mechanism of phagocytosis, and this process is primarily mediated via several pathways (**Figure 2**): 1) Upon the capture the microbe by the pseudopodia of neutrophils and the subsequent formation of phagosomes, a segment of the plasma membrane invaginates, allowing the combination of cytochrome b558, one of the subunits of NADPH oxidase embedded in the plasma membrane, with another oxidase subunit resident in the cytoplasm. This juxtaposition culminates in the activation of NADPH oxidase, thereby initiating an oxygen-dependent cytotoxic process; 2) Phagosomes detach from the plasma membrane and enter the cytoplasm where they fuse with lysosomes that store cytotoxic proteins, peptides, and enzymes. This connection can cause degranulation and trigger non-oxidative damage.

### Degranulation

Cytoplasmic granules, which constitute an arsenal of cytotoxic agents, are a hallmark of neutrophils. According to the composition of matrix and membrane proteins, these particles can be divided into four different subgroups (**Table 1**): primary (azurophilic) granules, secondary (specific) granules, tertiary (gelatinase) granules, and secretory vesicles (SVs) (2, 21). Primary granules contain antimicrobial substances, including neutrophil elastase (ELANE), cathepsin G, and myeloperoxidase (MPO). Secondary granules contain phagocytic receptors (such as Fc receptors and complement receptors), NADPH oxidase complexes, and lactoferrin. And tertiary granules contain receptors and gelatinase (56, 59, 64). The composition of neutrophil granules is related to various diseases, including rheumatoid arthritis, bullous pemphigoid, and cancer (53). For example, CD18, primarily stored in tertiary granules with some present in secondary granules (57), is associated with liver metastasis in colorectal cancer (65). NE and MMP-9 contribute to tumor metastasis by degrading the basement membrane and releasing vascular endothelial growth factor (VEGF) to promote angiogenesis (2, 53, 57, 66). ELANE is a neutrophil-specific serine protease stored in the azurophil granules (53, 57, 67). Studies have found that neutrophils exert anti-cancer effects by releasing ELANE, which selectively triggers cancer cell apoptosis and supports CD8^+^ T cell-mediated destruction of distant metastasis (68). ELANE selectively kills different types of tumor cells with minimal toxicity to non-cancer cells, which increases the possibility of developing it as a broad anti-cancer therapy. Degranulation, also referred to as exocytosis in neutrophils, relates to the release of pre-formed mediators from granules. The critical step in neutrophil degranulation is the fusion of granules with the plasma membrane, a process mediated by soluble N-ethylmaleimide-sensitive factor activating protein receptors (SNARE) (2, 67). Subsequently, the concentration of Ca^2+^ in neutrophils increases, triggering the degranulation of neutrophils (53, 69).

**Table 1:** Characteristics of neutrophil granules.

Granule	Biomarker	Representative substances	Time of formation	Content
primary granule	CD63, CD68, Granulophysin	peroxidase(+)	promyelocyte stage	acid phosphatase (67), MPO (2, 53, 57, 67), ELANE (53, 57, 67), cathepsin G (53, 57, 67), PRTN3 (53, 67)
secondary granule	CD15, CD66, CD67, CD11b/CD18, Gp91phox/p22phox	lactoferrin	myelocyte-metamyelocyte stage	FcR (46), CR (46), NADPH oxidase complex (2), lactoferrin (2), defensins/phagocytin (2), lysozyme (2, 67), NGAL (57), cyt-B (67)
tertiary granule	CD11b/CD18	gelatinase	band cell stage	Gelatinase (57), CD18 (57, 67), lysozyme (2, 67), cyt-B (67), MMP9 (57)
secretory vesicle	CD10, CD13, CD45, CR1, CD35	alkaline phosphatase	the late maturation of neutrophil	cyt-B (67), CR (67), plasma protein (2, 67)

### Oxygen burst

During the respiratory burst, superoxide anions catalyze the formation of ROS (70), including hydrogen peroxide (H_2_O_2_), hydroxyl radicals (OH·), and alkoxyl (RO·). Enormous evidence suggests that these oxidants play a crucial role in the clearance of tumor cells. The sudden increase of ROS can cause cell lysis necrosis, apoptosis, or other cell death pathways, so many studies emphasize the role of using ROS to promote oxidative death of cancer cells (71, 72). Traditional antitumor methods such as radiotherapy and chemotherapy, as well as emerging methods such as photodynamic therapy, are all aimed at increasing ROS levels to selectively eradicate malignant cells. Neutrophils can also inhibit lung metastasis and colonization by generating H_2_O_2_ (73).

ROS can modulate the protein conformation within tumor cells, thereby indirectly affecting the function of crucial signal transduction enzymes, such as kinases and phosphatases. This intricate interplay between ROS and signal transduction proteins forms the cornerstone of tumor therapeutic mechanisms. ROS can activate the intracellular signaling pathways of tumor cells, leading to the opening of non-selective cation channels transient receptor potential of melastatin 2 (TRPM2), which in turn causes lethal calcium influx into the cells (10). ROS can also disrupt the Ras-PI3K Akt signaling cascade by oxidizing specific cysteine residues in Ras and PI3K, thereby inhibiting their interaction and subsequently activating signaling pathways that drive tumor proliferation (74). The impact of ROS is not limited to the PI3K Akt signaling axis; It can also regulate the activity of its upstream signaling molecules, including receptor tyrosine kinase (RTK), ultimately leading to the death of tumor cells (75). In breast cancer cells, ROS can undermine the protein-protein interactions between Bcl-2 and apoptosis-promoting members of the Bcl-2 family, such as Bax and Bak (76), thereby fostering the production of apoptotic pores within mitochondrial membranes. This results in the release of Cytochrome c and the initiation of caspase cascade reactions, ultimately leading to the apoptosis of tumor cells (74).

Nevertheless, the levels of ROS are not static and can fluctuate drastically throughout the various stages of cancer progression. For example, in the early stages of cancer, an increase in ROS levels may accelerate cancer cell death, whereas in the advanced stages, targeting the antioxidant defense systems that permit cancer cells to prosper under elevated ROS conditions may be more effective. This dynamic change indicates that targeting ROS requires different strategies tailored to specific stages of cancer.

### NETs

Activated neutrophils can manufacture extracellular structures called NETs, which play an important role in capturing and eliminating microorganisms. These NETs are constituted of DNA, originating from either the nucleus or mitochondria (66), and a variety of proteins. Mass spectrometry has delineated 24 proteins associated with NETs, predominantly characterized by their cationic nature, among which are NE, citrullinated H3 histones, defensins, PRTN3, cathepsin G, and MPO (22). The implications of NETs in the context of cancer remain a subject of debate. Research demonstrated that tumor cells can stimulate neutrophils to form NETs through suicidal NETosis (77), and use it to facilitate the tumor metastasis (78). Recent studies also elucidated that NETs can directly attract tumor cells through the cell surface DNA sensor CCDC25 (79). ITGαvβ1, a component of NETs, can cause an epithelial mesenchymal transition in cancer cells, thereby promoting tumor cell chemoresistance (80). NETs can impede the interaction between immune cells and tumor cells by encapsulating the latter, thereby shielding them from the cytotoxic effects mediated by CD8^+^ T cells and natural killer (NK) cells (22). On the contrary, our previous research indicated that in patients with non-small cell lung cancer, NETs have the potential to hinder the migration of tumor cells and lead to their destruction by neighboring neutrophils (19). Recent studies have clarified that activated neutrophils in pancreatic ductal adenocarcinoma patients release NETs, where arginase 1 (ARG1) interacts with cathepsin S to obtain enzyme activity, thereby exerting anticancer effects (81).

## The tumor promoting effect of neutrophils

Neutrophils have inherent flexibility in adapting to environmental signals and are not limited by their initial maturation stage (42). Immature and mature neutrophils undergo reprogramming at the levels of epigenome, transcriptome, and proteome to support tumor growth after entering the tumor (27).

Neutrophils induce genomic instability by releasing ROS and RNS. The two active molecules ROS and RNS not only make DNA mutate directly, but also exacerbate DNA damage through the interaction of transcription factors like NF-κB and STAT3, hypoxia-inducible factor 1α (HIF-1α), kinases, growth factors, and cytokines (53, 82, 83). Nonetheless, there are also ROS-independent DNA damage and genetic instability mechanisms in neutrophils. For example, in inflammatory colon disease, activated neutrophils can stimulate double-stranded DNA break in intestinal epithelial cells by releasing vesicles containing pro-inflammatory microRNAs such as miR-23A and miR-155 (84).

Neutrophils can activate signaling pathways related to tumor cells, promoting tumor progression. In lung tissue damaged by radiation, the presence of neutrophils activates the Notch signaling pathway in metastatic tumor cells, which is a dominant factor in the self-renewal of cancer stem cells and the enhancement of tumorigenesis (85). Furthermore, tumor-infiltrating neutrophils use aconitate decarboxylase 1 (Acod1) to make itaconate, which regulates Nrf2-dependent ferroptosis and leads to the metastasis of breast cancer to the lungs by up-regulating the GM-CSF-JAK/STAT5-C/EBPb pathway (86).

Neutrophils also provide cellular contents that promote the proliferation and metastasis of cancer cells. Neutrophils pre-store an array of soluble mediators within granules—including enzymes, cytokines, and chemokines—to communicate with nearby cancer cells intercellularly, thus playing an assisting role in the process of metastasis by the matrix degradation (53, 60). In lung adenocarcinoma, ELANE infiltrates the tumor cells in the endothelium, leading to the down-regulation of insulin receptor substrate-1 (IRS-1), resulting in the enhancement of the interaction between phosphatidylinositol 3-kinase (PI3K) and the potent mitogen platelet-derived growth factor receptor (PDGFR), which eventually increase its proliferation ability (87). In addition, lung mesenchymal cells (MCs) promote the lipid storage in neutrophils or reprogram neutrophils to immunosuppressive phenotype by PGE_2_, then promote the breast cancer metastasis, and the MCs (88). Julia et al. demonstrated that neutrophils can form microtentacles (McTNs), allowing neutrophils to form heterotypic clusters with tumor cells, thereby promoting the reattachment, retention in distant sites during metastasis and formation of tumor cell clusters of circulating tumor cells during metastasis (89).

Metabolic reprogramming or dysregulation is a typical feature of cancer, and alterations in iron metabolism are considered a key factor driving the aggressive behavior of cancer cells (90), including unlimited proliferation, resistance to apoptosis, and enhanced metastatic potential. Pathologically activated neutrophils in the TME can exert immunosuppressive effects through ferroptosis, thereby reducing the effectiveness of T cell anti-tumor response (91). Neutrophil gelatinase-associated lipocalin (NGAL) could deliver iron to rapidly proliferating tumor cells, which not only supports tumor growth but also enhances its resistance to treatment, further highlighting the core role of neutrophils in the TME (90).

## Interactions between neutrophils and other immune cells

Neutrophils play a complex role in the TME, contributing both to immunosuppression and antitumor immunity. Initially, they can promote an immunosuppressive state by influencing various immune cells (21, 59, 60). Neutrophils secrete cytokines that negatively regulate diverse innate immune cells, thereby enhancing immunosuppression. They also utilize MPO and ROS to inhibit the function of NK cells, which consequently extends the survival of esophageal tumor cells (92). Furthermore, neutrophils impair the function of adaptive immune cells, such as CD8^+^ T lymphocytes, through multiple pathways (93). They engage in metabolic regulation utilize immune checkpoints, and exert specific molecular mechanisms to suppress the antitumor capabilities of immune cells. For example, tumor-associated neutrophils (TANs) consume essential metabolites like L-arginine, L-cysteine, and L-tryptophan that are necessary for T cell proliferation (48, 94, 95). Aging neutrophils can inhibit the stemness and tumor killing function of CD8^+^ T cells in elderly mice, thereby promoting tumor progression (96). Additionally, neutrophils can express ligands for inhibitory receptors like LOX1, CD84, and junctional adhesion molecule-like (JaML), which contribute to T cell depletion (22, 48). PD-L1-expressing neutrophils can also modulate the suppressive functions of T and NK cells through the PD-1/PD-L1 axis, thereby not only exacerbating immune suppression but also promoting tumor growth and progression (97, 98).

Paradoxically, neutrophils can also facilitate antitumor responses by orchestrating the recruitment and activation of immune cells, including macrophages, dendritic cells (DCs), NK cells, T lymphocytes, and B lymphocytes (**Figure 2**). They are integral in T cell-dependent antitumor immune networks and stimulate adaptive immune responses that inhibit the growth of malignancies. For instance, activated T cells in lung cancer increase the expression of co-stimulatory molecules on the neutrophil surface, enhancing T cell proliferation in a positive feedback loop (99). NETs can lower the activation threshold of T lymphocytes, thereby increasing their reactivity to specific antigens (100). In a melanoma model, T cells targeting melanoma cells expressing Trp1 trigger a secondary tumoricidal response by neutrophils, leading to the complete eradication of melanoma cells (101). Neutrophils also enhance the antigen-presenting capabilities of DCs and can act as antigen-presenting cells themselves, cross-presenting antigens to T cells and initiating antitumor immune responses (43). Leucine affects the epigenetic status of neutrophils through its metabolites, thereby regulating the antigen presentation ability of neutrophils, enabling them to activate T cells and improve the efficacy of tumor immunotherapy (102). Following antigen presentation, they directly influence the B cell response (55). Moreover, neutrophils produce various chemokines, including CXCL1, CXCL2, CXCL10, CCL2, and CCL3, which help recruit other immune cells and facilitate intricate bidirectional interactions (45). For instance, TANs have a higher ability to activate CCL4 transcription and recruit macrophages by secreting CCL4 (98). Additionally, neutrophils can induce macrophages to secrete interleukin-12 (IL-12), promoting INF γ pathway activation in unconventional αβ T cells (UTC_αβ_) and exerting antitumor functions during the early phase of sarcomas (60, 103).

This multifaceted role of neutrophils in the TME underscores their strategic importance in both promoting and suppressing tumorigenic processes.

## Prospective therapies targeting tumor-associated neutrophils

In the intricate background of TME, neutrophils play complex and multifaceted roles, which has sparked strong interest in developing therapeutic strategies for tumor immunotherapy targeting these cells. Approaches include neutrophil depletion, the inhibition of specific signaling pathways, and the modulation of neutrophil metabolism. The primary goal of such strategies is to selectively suppress pre-tumor neutrophils while enhancing the efficacy of antitumor neutrophils. The use of immune checkpoint inhibitors in combination with neutrophil-targeted therapies holds promise for enhancing antitumor immunity.

### Neutrophils depletion

Evidence from rodent cancer models has elucidated the potential benefits of neutrophil-targeted depletion therapies (45, 98), with the use of anti-Ly6G monoclonal antibodies (mAbs) demonstrating a selective consumption of neutrophils.

An agonist antibody against TRAIL-R2, DS-8273a, has been evaluated in patients with advanced cancers, showing a reduction in MDSCs in the peripheral blood of half the patients without affecting the maturation of myeloid cells and lymphocytes (45). Furthermore, the engagement of CD300ld, a biomarker of PMN-MDSCs, has been shown to modulate the cellular composition of the TME. Knockout and competitive blockade of the extracellular domain (ECD) of CD300ld can reverse the protumor effect of PMN-MDSCs (104). Ruxolitinib is a JAK inhibitor that has been shown in phase I clinical trials to significantly reduce the number of immature neutrophils without affecting the production of normal neutrophils, thereby achieving anti-tumor effects (105). However, in humans, depletion of neutrophils increases the risk of invasive infections, which may lead to increased treatment costs, antibiotic use, prolonged hospitalization, reduced or delayed use of chemical drugs, and in severe cases, life-threatening complications such as septic shock and sepsis syndrome, and even patient death. In clinical practice, G-CSF is often used preventively in cancer patients to prevent adverse consequences caused by excessive reduction of neutrophils (106).

### Immune checkpoint combination therapy

The cytotoxic potential of neutrophils is regulated by a delicate balance of signals transmitted through immune checkpoints and activating receptors. Neutrophils also express a suite of lymphocyte checkpoint ligands, including V-domain Ig suppressor of T cell activation (VISTA), PD-L1, CD86, 4-1BB ligand (4-1BBL), and OX40L (22), signaling a new frontier in cancer immunotherapy through targeted neutrophil checkpoints.

Recent findings suggest that targeting NAMPT can inhibit SIRT1 signaling and neutrophil angiogenic gene transcription, offering a potential mechanism for tumor growth inhibition (12). ATG-019, which inhibits both PAK4 and NAMPT, has shown improved efficacy in combination with anti-PD-1 therapy in murine tumor models compared to anti-PD-1 alone, representing a novel treatment strategy for advanced solid tumors and non-Hodgkin’s lymphoma (107, 108). Additionally, the concurrent administration of TNF and anti-CD40 mAb boosts neutrophil activation and toxicity, further promoting tumor cell apoptosis and clearance by enhancing oxidative damage and N1 neutrophil recruitment in the TME (13, 101). Additionally, the anti-CD40 therapy increased the Sell^hi^ neutrophils accumulation and maturation, thus eliciting the neutrophils antitumor response (109).

Compared to traditional radiotherapy and chemotherapy, immune checkpoint inhibitors typically have a lower incidence of adverse reactions. Even if patients stop using immune checkpoint inhibitors, they can often still maintain the treatment benefits (110). However, immunotherapy also faces the challenge of drug resistance. Evidence suggests that the binding affinity of anti-PD-1 receptors decreases within 2-3 months after the last dose of medication (111). In addition, multiple types of tumors and most patients have limited response to single immune checkpoint therapy, and combining other antitumor therapies may be the future path for immune checkpoint inhibitors.

### Blocking neutrophil recruitment to primary tumors and metastatic sites

Traditional strategies typically involve depleting the original tumor neutrophils or disrupting their migratory ability, rather than eliminating the entire neutrophil population. As previously noted, CXCR2 and CXCR1 are indispensable for the recruitment of neutrophils into the TME (21). The inhibition of neutrophil recruitment by blocking CXCL8, CXCR1, and CXCR2 has progressed to the clinical evaluation stage (22). The spleen serves as a reservoir for the precursors of TANs, from which TANs are recruited into the tumor stroma by CXCL8 (48, 49). Under normal physiological conditions, CXCL8 is barely detectable; however, within the TME, it rapidly accumulates to effectively regulate the protumor effects of N2 neutrophils, including the promotion of angiogenesis, dedifferentiation, and metastasis of tumor cells (50). The lipid metabolism-related gene enoyl-CoA δ-isomerase 2 (ECI2) can reduce CXCL8 to decrease neutrophil infiltration and NETs formation in the TME, thereby inhibiting colorectal cancer progression (112). Similarly, active PRSS35 can inactivate and degrade CXCL2 by cleaving it, ultimately inhibiting the development of HCC through a similar pathway (113). Inhibiting CXCL8 signal transduction can reduce the recruitment of N2 neutrophils. HuMax-IL8 (BMS-986253), an inhibitor of CXCL8, has been demonstrated to be well-tolerated in patients with advanced cancers and is currently being evaluated for its safety and efficacy in combination with nivolumab (NCT03400332) (50, 114). ABX-IL8, functioning as a neutralizing antibody against CXCL8, can significantly inhibit the promoter activity and collagenase activity of MMP2 in melanoma cells, thereby diminishing tumor metastasis, reducing tumor angiogenesis, and augmenting tumor apoptosis (50).

Lactate constitutes an effector molecule that not only contributes to inflammation but also plays a crucial role in the onset and progression of cancer (115). Consequently, lactate dehydrogenase inhibitors may represent effective targets for inhibiting tumor progression. Such inhibitors have been proven to alleviate the harmful neutrophil mobilization associated with TAN in malignant tumors (115).

### Reduce NETs formation

While NETosis plays an important role in microbial defense, it may be adeptly subverted by tumors to serve their own benefit (59). NETs not only inhibit T cell function but also facilitate the metastasis of tumor cells. By forming a physical barrier on the tumor’s surface, NETs shield tumor cells from CD8^+^ T cell-mediated assaults (116). Moreover, NETs can induce CD4^+^ T cells to differentiate into regulatory T cells (Treg cells), thereby establishing a tumor tolerant environment (39). As an emerging target for tumor therapy, the employment of deoxyribonuclease I (DNase I) to clear NETs or the use of related metabolic inhibitors can effectively repress NETosis (21). DNase I has been shown to have the ability to prevent the invasion and migration of lung and colon cancer cells, as well as significantly diminish lung metastasis in tumor models (22, 53). In addition, metformin targeting the mitochondrial DNA (mtDNA) of NETs can alleviate metastasis-abetting inflammatory state, thereby weakening the metastatic potential of hepatocellular carcinoma. The NET inhibitor chloroquine can alleviate hypercoagulability by diminishing NET-dependent thrombosis in cancer patients (39).

The citrullination of histones by peptidylarginine deiminase 4 (PAD4) is considered the main event in the formation of NETs *in vivo* (21, 46, 59). PDA4 inhibitors can enhance the clinical prognosis for tumor patients by diminishing NETosis (117). Consequently, PAD4 is expected to become an apt target for therapeutic intervention (66). The currently used PAD4 inhibitor in clinical practice is Cl-amidine (21). Moreover, other PAD4 inhibitors, such as BMS-P5, JBI-589, resveratrol (RES), and Simvastatin, have also exhibited therapeutic efficacy in animal models (118).

The formation of NETs is contingent upon H_2_O_2_ produced by NADPH oxidase and further metabolized by MPO (2). Therefore, ROS are amongst the most significant inducers of NETs (46, 53, 59), and any substance capable of inhibiting ROS production holds the potential to inhibit NETosis. Previous studies revealed that vitamin C, flavonoids, 5-aminosalicylic acid (5-ASA), N-acetyl-L-cysteine (NAC), PF-1355, or diphenyliodonium (DPI) can inhibit ROS production, thereby suppressing NETosis (21, 118). However, this aspect remains unexplored in the context of tumor treatment.

Although numerous studies have demonstrated that they have a pro-tumor effect, there are also studies suggesting that NETs can inhibit tumor growth by capturing and killing cancer cells (19, 81). In clinical practice, it is crucial to identify the role that NET plays in tumor progression.

### Target neutrophil metabolism

The immunomodulatory characteristics of neutrophils within TME are closely related to metabolic remodeling. In response to the increased energy demand caused by rapid proliferation of tumor tissue, neutrophils are adept at utilizing glucose, amino acids, and lipids as key energy substrates within the TME (119). Notably, TANs predominantly obtains energy from glycolytic metabolism, allowing them to function effectively in hypoxic conditions (42, 59). The hypoxic-glycolytic niche in tumors can reprogram mature and immature neutrophils into long-lived and terminally-differentiated subsets, thereby promoting angiogenesis and tumor growth (120). HIF-1α, a pivotal transcriptional regulator, control the expression of enzymes related to glycolysis and facilitates a metabolic shift from oxidative phosphorylation (OXPHOS) to glycolysis in PMN-MDSCs (48). *In vitro* experimentation with PX-478, an inhibitor of HIF-1α, has demonstrated synergistic enhancement of tumor cell apoptosis when used in conjunction with immune checkpoint inhibitors (121). Despite being highly dependent on glycolysis, neutrophils have the metabolic versatility to eschew glycolysis and upregulate alternative metabolic pathways, including glutaminolysis, OXPHOS, and fatty acid oxidation (FAO), when necessary (22, 60, 119). This metabolic adaptability highlights the challenge of targeting TAN metabolism within TME. Findings have elucidated that immunosuppressive neutrophils present within the TME elevate the expression of lipid transport-related proteins, including CD36 and fatty acid transporter protein 2 (FATP2) (22). The increase in CD36 expression promotes the metabolic transition from glycolysis to FAO, making FAO the main energy source for PMN-MDSC (95). Meanwhile, FATP2 promotes tumorigenesis and immune evasion by regulating the accumulation of arachidonic acid in PMN-MDSC and subsequent synthesis of prostaglandin E_2_ (PGE_2_) (48, 122). Consequently, FATP2 and CD36 have emerged as viable targets for selective inhibition to enhance the therapeutic efficacy of cancer treatments (95, 122). The metabolic profiling of TANs critically influences their behavior during tumorigenesis. The metabolic remodeling undergone by neutrophils within neoplastic tissue may provide novel avenues for research and development within future cancer treatment strategies.

## Other approaches to inhibit neutrophil function

### Coley’s Toxin and the BCG

The administration of Coley’s Toxin and the BCG vaccine represents one of the pioneering instances of immunotherapy in modern medical practice. The therapeutic essence of both Coley’s Toxin and the BCG vaccine lies in their capacity to stimulate neutrophils and other components of the immune system through the introduction of inactivated or attenuated bacterial agents (123, 124). Coley’s Toxin can trigger the release of a series of immune regulatory mediators, including cytokines, lysosomal enzymes, and ROS, which play an important role in directly killing tumor cells and activating other immune effectors (such as NK cells and T lymphocytes) to participate in synergistic antitumor responses (123). Similarly, early investigations have revealed that neutrophils activated by BCG immunotherapy in the context of bladder cancer undergo polarization into an antitumoral phenotype (14). The cytokines released by neutrophils activated by BCG vaccine indirectly enhance the chemotaxis of T cells, thereby enhancing the efficacy of BCG immunotherapy (125).

Applying microbial bioparticles to tumor growth not only activates the killing ability of neutrophils, but also causes significant changes in their transcriptional landscape, migration patterns, and functional properties, ultimately inhibiting tumor proliferation in a neutrophil-dependent manner. The intratumoral delivery of microbial bioparticles has also been observed to enhance the expression of chemokines recruiting NK cells and CD8^+^ T cells in neutrophils, thereby enhancing the antitumor immune response (126).

### Tumor cell-derived drug-loaded microparticles

**Figure 3:**
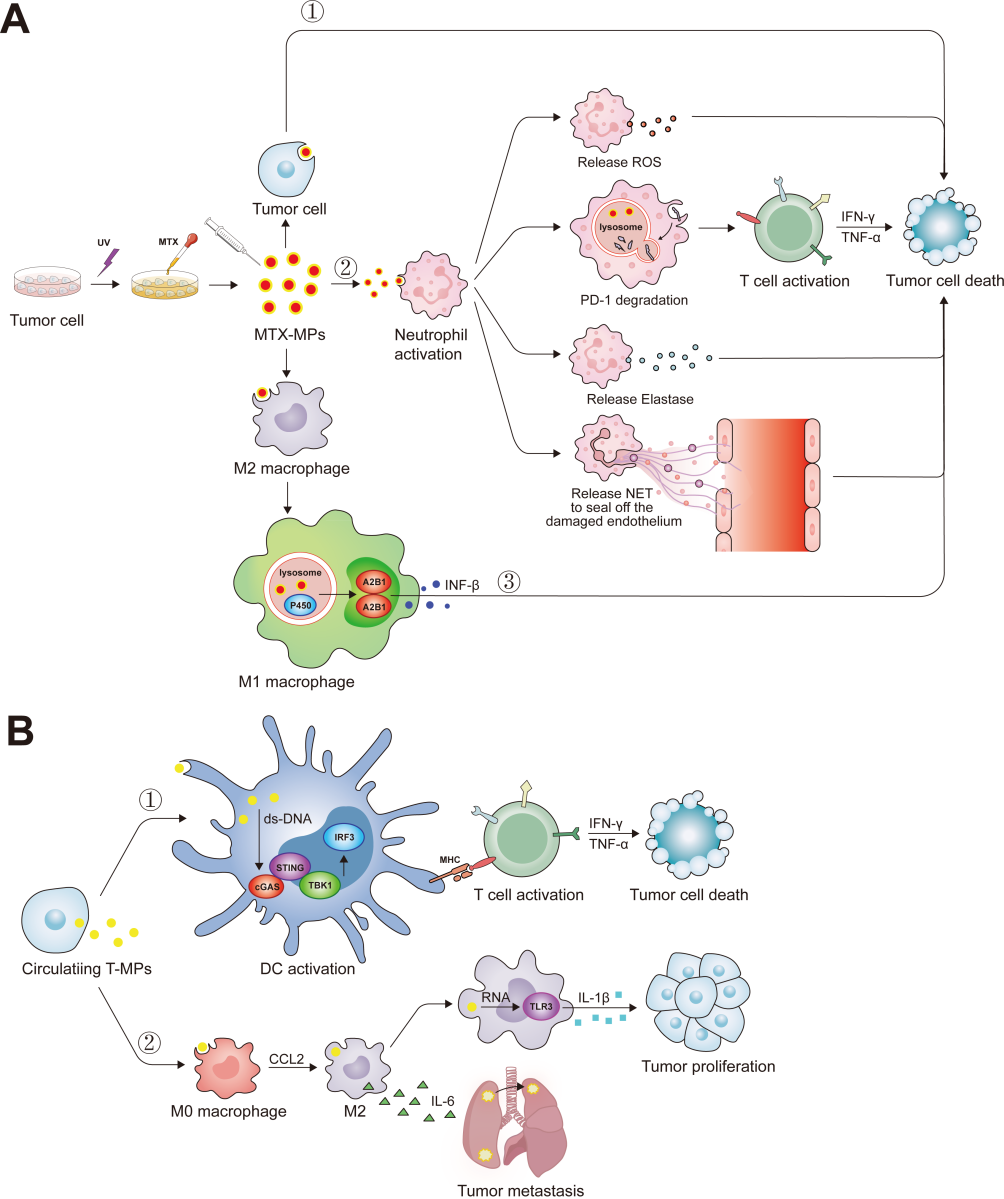
The biological function of tumor microparticles. **(A)** Ultraviolet radiation has the potential to elicit apoptosis in tumoral cells, facilitating the acquisition of MTX-MP complexes. Upon administration into the organism, these MTX-MPs are capable of deploying antineoplastic actions through a variety of mechanisms: ①Directly inducing apoptosis after tumor cell ingesting MTX-MPs. ②Upon MTX-MPs uptake by neutrophils, they stimulate the neutrophils’ antitumor functions, which include the emission of ROS, the enhancement of T-cell-mediated antitumor responses through the degradation of PD-1 via the lysosome, the secretion of Elastase, and the extrusion of NETs to repair the endothelial layer. ③MTX-MPs are instrumental in prompting the polarization of M2 macrophages into the M1 phenotype. Subsequent to this transformation, M1 macrophages promote the secretion of INF-β through the lysosomal cytochrome P450 and nuclear hnRNPA2B1 signaling pathways. **(B)** Tumor cells also circulate and transport T-MPs to the periphery during their progression. ①These T-MPs harbor double-stranded DNA of tumor origin, which, upon internalization by DCs, activates the cGAS-STING pathway, markedly augmenting the DCs’ capacity for antigen presentation. ②Following the ingestion of T-MPs, M0 macrophages undergo polarization into M2 macrophages, and the non-coding RNA present within the T-MPs triggers the activation of TLR3, resultant in an augmented secretion of IL-1β by the macrophages and a concomitant promotion of tumor cell proliferation. Moreover, M2 macrophages secrete IL-6, thereby fostering the pulmonary metastasis of lung cancer cells.

Previous studies have shown that tumor cells can release various types of extracellular vesicles, one of which is called tumor microparticles (T-MP) with sizes ranging from 100-1000 nm (127). T-MPs have been shown to affect various cancer-related cells and immune cells (**Figure 3**). As a carrier for chemotherapeutic drug delivery, MP has the advantages of biological safety, simple preparation, and stable properties. The MPs deliver the chemotherapeutic drugs to the nucleus and kill the tumor repopulation cell (128). In addition, the MP contains the tumor common antigen, which activated the DC by cGas-STING pathway, then promotes the tumor-specific CD8^+^ T cells antitumor response (129). Our previous studies have clarified that methotrexate-loaded tumor cell microparticles (MTX-MPs) activated neutrophils and demonstrated a high level of efficacy and safety in a clinical trial involving patients with MPE or cholangiocarcinoma (18, 19). Recently, we found that the MTX-MP-activated neutrophils promote the T cell antitumor response by increasing the PD-1 degradation in the lysosome pathway in neutrophils (16). Moreover, the MPs without drug promote tumor metastasis by promoting M2 macrophage differentiation, while the MTX-MP promotes M2 polarized toward M1 by activating lysosomal cytochrome P450 and nuclear hnRNPA2B1, thereby recruiting neutrophils to kill tumor cells (17, 129, 130).

### Trained immunity

For a long time, people have believed that immune memory is a unique feature of adaptive immune response. Professor Mihai Netea from Nijmegen University first proposed that the innate immune system can also exhibit adaptive features, known as trained immunity (131). One of the most typical examples is that the BCG can induce the well-trained immunity of neutrophils through the genome-wide epigenetic modifications in trimethylation at histone 3 lysine 4 (132). β-glucan can train the innate immune system through type I interferon signaling, reprogram GMP, and reprogram neutrophils into antitumor phenotypes, ultimately inhibiting the occurrence and development of tumors (133). Mulder et al. designed a bone marrow-targeted nanobiological agent that can induce training immunity, thus enhance proliferation and metabolism of neutrophils to inhibit tumor growth (134).

## Conclusions

Despite commendable progress in understanding the biological complexity, functional characteristics, and heterogeneity of neutrophils, their critical role and significance in the context of cancer have long been overlooked. However, in the past decade, there has been increasing interest in neutrophils in TME. On one hand, neutrophils can contribute to genetic instability within tumor cells, promote angiogenesis, and inhibit the antitumor responses of other immune cells. On the other hand, they can exert antitumor effects through phagocytosis, the production of ROS, and the activation of adaptive immune responses. The dual function of neutrophils in tumor immunity depends on their diversity and plasticity, and the seemingly paradoxical nature of these roles may well be attributable to differential influences exerted by the TME on neutrophil maturation, function, and polarization. So far, most studies have focused on the pre tumor function of neutrophils; however, it remains to be explored whether it is feasible to modulate and harness antitumor capacities.

In summation, a comprehensive understanding of the distinctive characteristics, recruitment processes, and functions of neutrophils within the TME is crucial for developing effective tumor immunotherapy strategies. Further research is needed to elucidate the mechanisms underlying the dual role of neutrophils in tumor biology and to investigate the therapeutic potential of targeting neutrophils in cancer treatment.
